# The moderated mediation effect of physical activity on non-suicidal self-injury: the mediating role of perceived stress and the moderating role of parental support

**DOI:** 10.3389/fpsyt.2026.1824693

**Published:** 2026-08-19

**Authors:** Pengyu Wang, Hui Jia, Jie Xu

**Affiliations:** 1 School of Public Administration, Henan University of Economics and Law, Zhengzhou, China; 2 Department of Marine-Sports, Pukyong National University, Busan, Republic of Korea; 3 Sichuan Technology and Business University, Meishan, China

**Keywords:** moderated mediation, non-suicidal self-injury (NSSI), parental support, perceived stress, physical activity

## Abstract

**Objectives:**

Non-suicidal self-injury (NSSI) has emerged as a significant public health challenge among adolescents globally. Although physical activity (PA) is widely recognized as a potential protective factor for mental health, the internal mechanisms through which it is associated with specific high-risk behaviors like NSSI remain insufficiently explored. This study aims to construct and validate a moderated mediation model to examine the association between PA and adolescent NSSI, the mediating role of perceived stress, and the boundary conditions set by parental support.

**Methods:**

Utilizing a cross-sectional observational design and a stratified cluster sampling method, 402 adolescents (198 males and 204 females; mean age = 15.23 ± 1.57 years) were recruited from five middle and high schools (three urban, two rural) in Sichuan Province, China. Participants completed a battery of psychometric instruments, including the Physical Activity Rating Scale-3 (PARS-3), the Perceived Stress Scale-10 (PSS-10), the Inventory of Parent and Peer Attachment (IPPA), and the Deliberate Self-Harm Inventory (DSHI). Data analysis was conducted using SPSS 26.0 and the PROCESS macro (Model 58).

**Results:**

PA was significantly and negatively associated with adolescent NSSI (β = -0.58, p < 0.001). Perceived stress partially mediated this relationship, with the indirect association accounting for 36.21% of the total effect. The substantial direct association (63.79%) suggests that PA may also relate to NSSI through other unmeasured pathways, such as speculative neurobiological adaptations or behavioral replacement. Furthermore, parental support significantly moderated the mediated process. Specifically, parental support moderated both the PA -> perceived stress pathway (β = 0.15, p = 0.003) and the perceived stress -> NSSI pathway (β = -0.14, p = 0.005), suggesting that the negative association between PA and stress is stronger under low parental support.

**Conclusions:**

PA serves as a potential protective factor negatively associated with NSSI through both direct and indirect statistical pathways. Parental support functions as a critical environmental buffer; for adolescents lacking parental support, PA serves as a crucial compensatory resource for stress management. These findings offer empirical evidence for the development of integrated family-school intervention strategies for adolescent mental health.

## 1 Introduction

Non-suicidal self-injury (NSSI) refers to the deliberate, intentional destruction of one’s own body tissue without suicidal intent, commonly manifesting in forms such as cutting, burning, scratching, or hitting (1, 2). In recent decades, NSSI has emerged as a critical public health issue in adolescent mental health worldwide. Epidemiological data indicate that the lifetime prevalence of NSSI among adolescents ranges from 17.7% to 22.1%, showing a significant upward trend (3). Between 2007 and 2023, the severity of adolescent NSSI increased by 0.35 standard deviations, representing a medium effect size. In China, the lifetime prevalence among adolescents has reached 24.7%, while research in Singapore reports rates as high as 25.0% (4). NSSI is not only closely associated with internalizing problems such as depression and anxiety (5) but also serves as a potent longitudinal predictor of future suicidal behavior; research suggests that over 66% of adolescents who attempt suicide have a history of NSSI (6). Given that NSSI typically emerges between the ages of 12 and 16 and peaks around age 15 (7), exploring modifiable protective factors and their underlying mechanisms is of significant scientific and practical importance for the formulation of early prevention strategies (8).

Physical activity (PA), as an accessible and cost-effective lifestyle intervention, has demonstrated great potential in promoting adolescent mental health. A recent systematic review and meta-analysis of 76 studies involving 3, 007 participants showed that PA interventions significantly benefit the overall mental health of children and adolescents (g=0.67), improving both internalizing (g=0.72) and externalizing problems (g=0.58) (9). However, existing research primarily focuses on the direct impact of PA on general psychological issues, while exploration of its relationship with specific high-risk behaviors such as NSSI remains relatively limited (10). However, recent empirical studies and reviews emphasize that PA is significantly associated with a reduced frequency of NSSI. For instance, Zhou et al. (2024) found that PA is related to lower NSSI through stress reduction among depressed adolescents, though the underlying psychosocial boundary conditions remain unclear (11). Furthermore, research suggests that PA may act as a protective mechanism against self-injurious impulses particularly in populations with affective vulnerabilities, highlighting the need to explore indirect cognitive and environmental pathways (12). Theoretically, PA may influence NSSI through two distinct pathways: a direct association by enhancing overall physical and mental well-being, and an indirect association by modulating stress-related psychophysiological processes (11).

Among the numerous risk factors for NSSI, perceived stress is identified as a core proximal trigger (13). The Stress-Diathesis Model suggests that when individuals face environmental demands that exceed their available coping resources, they may employ NSSI as a maladaptive strategy to regulate negative emotions or restore physiological homeostasis (14). Although the association between stress and NSSI is well-established, traditional direct-effect models fail to explain why not all high-stress individuals develop NSSI behaviors. This theoretical gap suggests the necessity of introducing moderating and mediating mechanisms to clarify the boundary conditions and process pathways through which stress transforms into NSSI. Furthermore, developmental psychopathology emphasizes that the occurrence of NSSI results from the dynamic interaction between the individual and their environment, requiring a focus on how modifiable protective factors buffer the influence of risk factors (15).

This study proposes that perceived stress mediates the relationship between PA and NSSI, while parental support moderates this mediation process, forming a “moderated mediation model.” This theoretical construction is grounded in three lines of evidence: First, the buffering mechanism of PA on perceived stress. According to the Cross-Stressor Adaptation Hypothesis, regular PA acts as an intermittent physiological stressor that optimizes the responsiveness of the hypothalamic-pituitary-adrenal (HPA) axis, thereby enhancing physiological resilience to psychosocial stress. Empirical research indicates that PA can effectively alleviate subjective stress experiences by lowering cortisol levels, improving HPA axis negative feedback regulation (16), reducing inflammatory responses, and promoting the expression of brain-derived neurotrophic factor (BDNF). Experience sampling method (ESM) studies further confirm that after engaging in PA, the positive correlation between current stress and negative affect is significantly weakened, demonstrating an immediate “stress-buffering” effect (17). Thus, PA may be associated with a reduced occurrence of NSSI by lowering perceived stress levels. Second, the mediating role of perceived stress in the occurrence of NSSI. The Emotional Cascade Model suggests that the cycle of rumination and negative emotions leads to heightened stress perception, which is a key mechanism triggering NSSI (18). In states of high stress, adolescents are more likely to utilize NSSI to quickly alleviate emotional pain or restore emotional stability. Longitudinal studies have shown that COVID-19-related stress significantly predicts the frequency of NSSI, a link that remains robust regardless of social support levels (19). Therefore, utilizing perceived stress as a mediator connecting PA and NSSI aligns with functional emotional regulation theories of NSSI behavior. Third, parental support as a key moderating variable. Parental support is the most central environmental protective factor in adolescent development. According to the StressBuffering Hypothesis, high-quality parental support can weaken the negative impact of stress on maladaptive behaviors by providing emotional security and coping resources (20). Furthermore, in the context of Chinese culture, where family cohesion and filial piety are heavily emphasized, parental support serves as the most fundamental psychosocial resource for adolescents navigating intense academic and social pressures (5). While some studies have failed to find a buffering effect of parental support on the stress-NSSI relationship, recent research indicates that matching patterns of parental and peer support can indirectly reduce NSSI by lowering bullying victimization (21). More importantly, according to the Conservation of Resources (COR) theory and the resource substitution hypothesis (22, 23), when individuals lack key external social resources (such as parental support), internal or alternative coping resources (such as physical activity) become disproportionately more critical for maintaining psychological homeostasis. Therefore, we hypothesize that parental support may moderate the “PA → Perceived Stress” pathway in a compensatory manner: under low parental support, the negative association between PA and stress is expected to be stronger, as PA acts as a crucial substitute coping resource; whereas under high parental support, the necessity of PA for stress reduction may be attenuated. This “moderated mediation” effect (24) has yet to be systematically examined in the field of adolescent NSSI.

In summary, this study aims to construct and verify a moderated mediation model to investigate the indirect associations between PA and adolescent NSSI and the moderating role of parental support. This model integrates psychological and social perspectives, expanding the research on the PA-NSSI relationship and providing an empirical basis for home-school joint intervention programs. By revealing how the “PA →Perceived Stress →NSSI” pathway varies with parental support levels, this study seeks to offer new theoretical insights into the interaction between modifiable risk and protective factors in NSSI prevention. Based on the integrated theoretical framework of the Stress-Diathesis Model and Conservation of Resources (COR) theory, the present study proposes the following hypotheses: Hypothesis 1 (H1), physical activity (PA) is significantly and negatively associated with adolescent NSSI; Hypothesis 2 (H2), perceived stress mediates the relationship between PA and NSSI; and Hypothesis 3 (H3), parental support moderates the mediated pathways. Specifically, the negative association between PA and perceived stress (H3a), and the positive association between perceived stress and NSSI (H3b), are expected to be stronger among adolescents with low parental support compared to those with high parental support, reflecting a compensatory mechanism.

## 2 Materials and methods

### 2.1 Participants and procedure

A cross-sectional observational survey design was employed using stratified cluster sampling. Participants were recruited from five representative middle and high schools in China, comprising three urban schools and two rural schools to ensure diverse socioeconomic and geographic representation. A total of 420 questionnaires were distributed during regular school sessions. After rigorous data cleaning—including the exclusion of incomplete responses, patterned answering (e.g., identical responses for all items), or surveys with missing data exceeding 5%—402 valid questionnaires were retained, resulting in an effective response rate of 95.71%. The final analytical sample consisted of 198 males (49.25%) and 204 females (50.75%). The age of participants ranged from 12 to 18 years, with a mean age of 15.23 ± 1.57 years. Regarding academic distribution, 226 participants (56.22%) were junior high school students, and 176 (43.78%) were senior high school students. Given the six-year age span and the inclusion of both junior and senior high school students, a preliminary sensitivity analysis was conducted. The results indicated that while senior high school students reported slightly higher mean stress levels, the direction and significance of the bivariate correlations among the core variables remained consistent across both developmental cohorts, thereby theoretically and statistically justifying the pooling of the sample for the primary analyses.

Weekly physical activity (PA) participation duration was distributed as follows: 189 participants (47.01%) reported less than 150 minutes, 145 (36.07%) reported 150–300 minutes, and 68 (16.92%) reported more than 300 minutes. A total of 98 participants (24.38%) reported having engaged in NSSI behavior at least once, a figure consistent with current epidemiological prevalence rates in Chinese adolescent populations. The study protocol was approved by the Professor Committee of Sichuan Technology and Business University (Approval No. STBU-PC-20250318) and adhered to the ethical standards of the Declaration of Helsinki. Prior to data collection, written informed consent was obtained from all students and their legal guardians (parents or caregivers). Participants were briefed on the voluntary nature of the study, their right to withdraw at any time without academic penalty, and the strict confidentiality of their personal data. All responses were anonymized for analysis.

### 2.2 Measurement instruments

Detailed questionnaire sources and acquisition procedures are provided in **Supplementary Material S1**.

#### 2.2.1 Non-suicidal self-injury

Adolescent NSSI was assessed using the Deliberate Self-Harm Inventory (DSHI) (25). The DSHI was selected because it provides a behavior-specific assessment of deliberate self-harm and has been adapted for adolescent samples, including studies using modified DSHI formats in 15-year-old adolescents (26). This 17-item self-report instrument evaluates the lifetime prevalence, frequency, and severity of various self-injurious behaviors (e.g., cutting, burning, scratching, hitting) without suicidal intent. The assessment followed a two-stage reporting logic: participants first indicated whether they had ever engaged in a specific behavior. If they answered “yes, “ they reported the frequency of that behavior over the past 12 months on a 7-point scale ranging from 0 (never) to 6 (more than 20 times). This 7-point scale is particularly suitable for capturing the wide variability of lower-frequency NSSI behaviors typical in community-dwelling youth, and was selected due to its high sensitivity in capturing these subtle variances. Total scores range from 0 to 102, with higher scores signifying greater severity and higher frequency of NSSI. In the current sample, the Cronbach’s α for the DSHI was 0.84, demonstrating robust internal consistency.

#### 2.2.2 Physical activity

The PARS-3 was selected due to its brevity, established cross-cultural reliability, and specific validation for evaluating exercise habits in Chinese adolescent cohorts (10). The Physical Activity Rating Scale-3 (PARS-3), revised for Chinese use by Liang (27), was used to quantify levels of physical activity (10). The scale measures three key components: intensity (e.g., “To what extent do you experience sweating or breathlessness during exercise?”), duration (e.g., “Average duration per exercise session?”), and frequency (e.g., “Frequency of exercise per week?”). Each item is rated on a 5-point scale. The total PA score is calculated using the established formula: Intensity×(Duration−1)×Frequency, resulting in a score range from 0 to 100. Based on these scores, activity is categorized into low (≤19), moderate (20–42), and high (≥43) levels. Despite its widespread use and high reliability (in this study, the Cronbach’s α was 0.79, and the McDonald’s ω was 0.81), we acknowledge that the PARS-3 relies on retrospective self-report, which may introduce recall bias. Additionally, its formula-based composite scoring matrix may obscure the independent measurement properties and linear contributions of specific exercise dimensions.

#### 2.2.3 Perceived stress

Subjective stress perception over the past month was evaluated using the 10-item Perceived Stress Scale (PSS-10), which derives from Cohen et al.’s original Perceived Stress Scale (28) and has shown satisfactory psychometric evidence in reviews and Chinese adolescent validation studies (29, 30). This instrument was chosen for its focus on recent cognitive appraisals of unpredictable stressors, which is highly relevant to adolescent academic life. The instrument utilizes a 5-point Likert system (0 = never to 4 = always) and consists of two sub-factors: perceived helplessness (6 items, e.g., “In the last month, how often have you felt that you were unable to control the important things in your life?”) and perceived self-efficacy (4 items, reverse-scored, e.g., “In the last month, how often have you felt confident about your ability to handle your personal problems?”). Total scores range from 0 to 40, with higher scores reflecting higher levels of subjective stress. In the present study, the Cronbach’s α for the total scale was 0.81, while the alphas for the subfactors were 0.83 (helplessness) and 0.78 (self-efficacy).

#### 2.2.4 Parental support

Adolescents’ perceived quality of parental support and attachment was measured using the Parent Scale of the Inventory of Parent and Peer Attachment (IPPA-45) (31). The 15-item short version measures three dimensions: trust (5 items, e.g., “My parents accept me as I am”), communication (5 items, e.g., “I tell my parents when I am angry”), and alienation (5 items, reverse-scored, e.g., “I feel my parents don’t understand me”). Items are rated on a 5-point Likert scale (1 = almost never to 5 = almost always). Higher total scores indicate higher perceived support and attachment security. The Chinese version of the revised IPPA has been validated in junior student samples and provides relevant support for its use with Chinese adolescents (32). In the current study, a confirmatory factor analysis (CFA) of the IPPA short form yielded a good model fit (χ2/df=2.45, CFI = 0.94, TLI = 0.92, RMSEA = 0.06), and the Cronbach’s α for the scale was 0.89, with sub-dimension coefficients of 0.85 (trust), 0.82 (communication), and 0.79 (alienation).

### 2.3 Data collection and quality control

The survey was conducted collectively in classrooms by six trained researchers. A standardized protocol was strictly followed, including uniform instructions to explain the survey objectives, anonymity, and response requirements. To mitigate the inherent recall bias associated with self-reported PA duration, rigorous quality control measures were implemented during administration. Researchers provided explicit reference anchors (e.g., standard 45-minute physical education classes, scheduled school running sessions) to help students accurately estimate their weekly exercise intensity and duration. Furthermore, extreme physiological impossibility outliers in PA reports were manually verified or excluded. To minimize social desirability bias, teachers were requested to leave the classroom during the administration. Upon completion, researchers conducted an immediate on-site review to identify and clarify obvious omissions. Data were digitized using a double-entry method to ensure accuracy. Prior to analysis, Z-score transformation was used to detect univariate outliers (|Z|>3.29), and missing values (less than 5% of total data) were handled using mean substitution.

### 2.4 Statistical analysis plan

Quantitative data analysis was performed using SPSS 26.0 and the PROCESS macro (Version 3.5) developed by Hayes (33). The analysis followed a multi-stage approach:

1. Common Method Bias (CMB): Considering the self-report nature of the data, Harman’s single-factor test was initially performed using unrotated exploratory factor analysis (EFA). To rigorously address the known limitations of Harman’s test, we supplemented this approach with a Confirmatory Factor Analysis (CFA) incorporating an Unmeasured Latent Method Factor (ULMF) to statistically evaluate whether method variance substantially distorted the measurement model.
2. Descriptive and Correlation Analysis: Mean (M), standard deviation (SD), and Pearson correlation coefficients were calculated to examine the distributions and bivariate relationships between core variables. To prevent multicollinearity resulting from the near-perfect correlation between participant age and school grade, grade was excluded from subsequent regression models. Given the wide age range (12–18 years), age and gender were entered as continuous and categorical covariates, respectively, in all PROCESS macro regression models. This statistical control isolates the variance attributed to developmental maturation, ensuring that the modeled pathways reflect the core psychological constructs independent of simple age effects. Variance Inflation Factors (VIF) were calculated to empirically confirm the absence of collinearity.
3. Mediation Analysis: PROCESS Model 4 was utilized to test the partial statistical mediation role of perceived stress (M) in the association between physical activity (X) and NSSI (Y).
4. Moderated Mediation Analysis: PROCESS Model 58 was employed to verify whether parental support (S) moderated both stages of the statistical mediation pathways, specifically the X→M (Physical Activity to Perceived Stress) and M→Y (Perceived Stress to NSSI) segments, consistent with moderated path analysis recommendations (34). Conditional indirect associations at low, medium, and high levels of the moderator were estimated, and the incremental variance explained (ΔR2) by the interaction terms was calculated to ascertain practical significance.
5. Effect Estimation: Bias-corrected bootstrapping with 5, 000 resamples was used to estimate the 95% confidence intervals (CI). Effects were deemed statistically significant if the CI did not cross zero. Demographic variables including gender and age were entered as covariates in all regression models to control for potential confounding effects.

## 3 Results

### 3.1 Common method bias test

Given that all variables were measured through self-report instruments, common method bias (CMB) was assessed using Harman’s single-factor test to ensure the validity of the statistical inferences. An unrotated exploratory factor analysis (EFA) was performed on all 52 items from the primary scales. The results, as summarized in **Table 1**, revealed 12 factors with eigenvalues greater than 1. The variance explained by the first principal factor was 28.76%, which is well below the critical threshold of 40%. Because Harman’s single-factor test has been criticized for its lack of sensitivity, we supplemented this approach with a Confirmatory Factor Analysis (CFA) to provide a more rigorous assessment. The hypothesized four-factor measurement model (Physical Activity, Perceived Stress, Parental Support, NSSI) demonstrated a good fit to the data (χ2/df=2.14, CFI = 0.94, TLI = 0.92, RMSEA = 0.05). By contrast, a single-factor model exhibited a very poor fit (χ2/df=9.87, CFI = 0.41, TLI = 0.38, RMSEA = 0.18). Furthermore, we introduced an Unmeasured Latent Method Factor (ULMF) to the four-factor model. The inclusion of the ULMF did not yield a significant improvement in model fit indices (ΔCFI<0.01, ΔRMSEA<0.01), and the standardized factor loadings of the primary traits remained significant. These findings suggest that common method bias does not pose a significant threat to the results of the current study.

**Table 1:** Harman’s single-factor test results.

Factor No.	Eigenvalue	Variance explained (%)	Cumulative variance (%)
1	10.354	28.76	28.76
2	5.218	14.49	43.25
3	3.872	10.75	54.00
4	2.965	8.24	62.24
5	2.153	5.98	68.22
6-12	1.021-1.897	2.84-5.27	100.00

### 3.2 Descriptive statistics and correlation analysis

The descriptive statistics, including means (M) and standard deviations (SD), along with the Pearson correlation coefficients for all core variables, are presented in **Table 2**.

**Table 2:** Descriptive statistics and intercorrelations among study variables (N = 402).

Variable	M ± SD	1	2	3	4	5	6	7
1. Gender	0.51 ± 0.50	1						
2. Age	15.23 ± 1.57	0.08	1					
3. Grade	0.44 ± 0.50	0.06	0.89***	1				
4. PA (X)	65.32 ± 21.45	-0.11	-0.09	-0.07	1			
5. Stress (M)	28.76 ± 6.89	0.15*	0.12	0.10	-0.38***	1		
6. Support (S)	52.38 ± 10.76	-0.08	-0.11	-0.09	0.29***	-0.42***	1	
7. NSSI (Y)	22.57 ± 8.92	0.18**	0.14	0.12	-0.58***	0.65***	-0.51***	1

Gender: 0 = Male, 1 = Female; Grade: 0 = Junior High, 1 = Senior High. *p < 0.05, **p < 0.01, ***p < 0.001.

As shown in **Table 2**, a near-perfect collinearity was observed between age and grade (r=0.89, p<0.001). To prevent multicollinearity from distorting the regression coefficients and inflating standard errors, grade was excluded from all subsequent regression analyses, retaining only gender and age as demographic covariates. Collinearity diagnostics were performed prior to model testing, confirming that the Variance Inflation Factor (VIF) for all predictors ranged from 1.05 to 1.45, well below the critical threshold of 10, indicating the absence of problematic multicollinearity.

After controlling for gender and age, the correlation analysis showed that physical activity (PA) was significantly and negatively correlated with both perceived stress (r=−0.38, p<0.001) and NSSI (r=−0.58, p<0.001). Perceived stress demonstrated a strong positive correlation with NSSI (r=0.65, p<0.001). Parental support was found to be positively associated with PA (r=0.29, p<0.001) and negatively associated with both perceived stress (r=−0.42, p<0.001) and NSSI (r=−0.51, p<0.001). These significant bivariate relationships provide the empirical prerequisite for testing the hypothesized mediation and moderation models.

### 3.3 Mediation analysis of perceived stress

To test the hypothesis that perceived stress mediates the statistical association between PA and NSSI, we utilized Hayes’ PROCESS macro (Model 4), controlling for demographic factors (gender and age). The results of the regression analysis are detailed in **Table 3**.

**Table 3:** Regression results for the mediation model of perceived stress.

Regression model	Predictor	β	SE	t	p	95% CI
Model 1 (Stress)	Controls	0.04	0.05	2.25	0.026	[0.01, 0.17]
Model 1 (Stress)	PA (X)	-0.35	0.05	-7.00	<0.001	[-0.45, -0.25]
Model 2 (NSSI)	Controls	0.05	0.06	2.40	0.017	[0.02, 0.22]
Model 2 (NSSI)	PA (X)	-0.58	0.06	-9.67	<0.001	[-0.70, -0.46]
Model 3 (NSSI)	Controls	0.10	0.04	2.50	0.013	[0.02, 0.18]
Model 3 (NSSI)	PA (X)	-0.37	0.05	-7.40	<0.001	[-0.47, -0.27]
Model 3 (NSSI)	Stress (M)	0.54	0.04	13.50	<0.001	[0.46, 0.62]

As shown in Model 1, PA significantly and negatively was associated with perceived stress (β=−0.35, p<0.001). Model 2 indicates that PA was a significant negative correlate of NSSI total association (β=−0.58, p<0.001). In Model 3, when both PA and perceived stress were included as predictors of NSSI, perceived stress was a significant positive predictor (β=0.54, p<0.001), while the direct association of PA with NSSI remained significant but was reduced in magnitude (β=−0.37, p<0.001).

**Figure 1:**
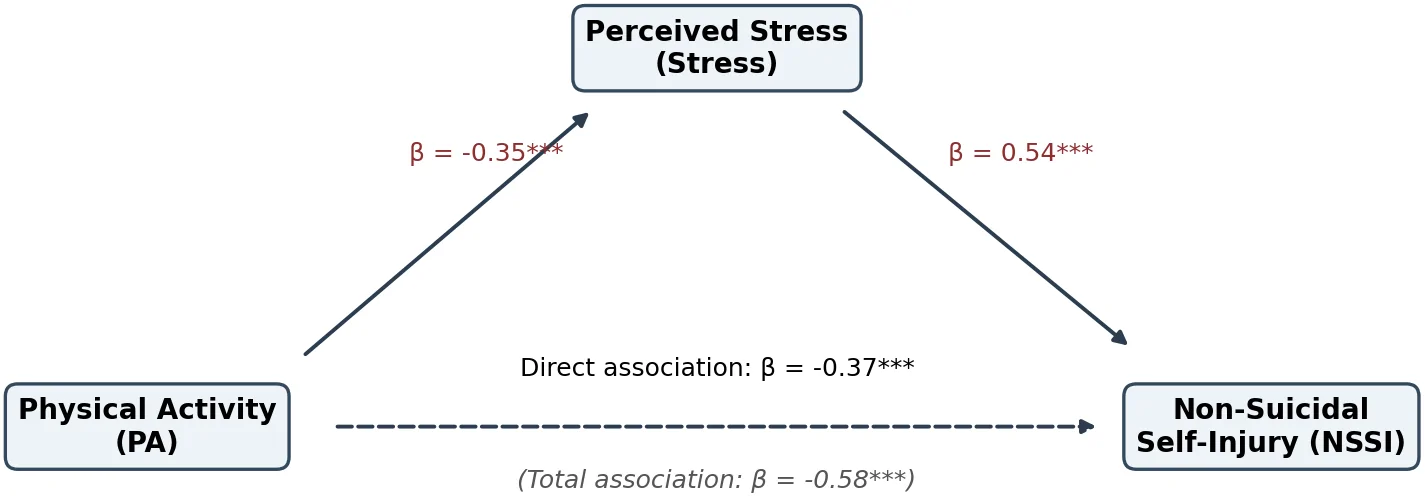
Standardized path coefficients of the mediation model. This diagram visualizes the mediating role of perceived stress. All paths are significant at p<0.001. Standardized coefficients are presented. Causal inferences cannot be drawn from these cross-sectional statistical pathways.

The decomposition of the statistical pathways using bias-corrected bootstrapping (5, 000 resamples) is presented in **Table 4**. The total association of PA on NSSI was -0.58. The indirect association through perceived stress was -0.21 (95% CI = [-0.29, -0.13]), which accounted for 36.21% of the total statistical effect. It is important to note that the direct association (-0.37) accounted for the remaining 63.79% of the total association, suggesting that while perceived stress is a significant psychological mediator, physical activity may also be linked to reduced NSSI through other substantial unmeasured mechanisms (e.g., speculative neurobiological adaptations or behavioral replacement). **Figure 1** provides a visual representation of this partial mediation model, illustrating the standardized path coefficients.

**Table 4:** Total, direct, and indirect effects decomposition.

Effect path	Effect size	SE	Boot 95% CI	Percentage (%)
Total Effect (X -> Y)	-0.58	0.06	[-0.70, -0.46]	100.00%
Direct Effect (X -> Y)	-0.37	0.05	[-0.47, -0.27]	63.79%
Indirect Effect (X -> M -> Y)	-0.21	0.04	[-0.29, -0.13]	36.21%

### 3.4 Moderated mediation analysis

To determine the boundary conditions of the mediation process, we conducted a moderated mediation analysis using PROCESS Model 58. This model simultaneously examined the moderating effect of parental support on both the PA →Stress and Stress →NSSI pathways. The regression parameters for this comprehensive model are summarized in **Table 5**.

**Table 5:** Regression models for the moderated mediation effect.

Outcome variable	Predictor	β	SE	t	p	95% CI	R²	F	ΔR²
Stress (M)	Controls	0.08	0.04	2.00	0.046	[0.00, 0.16]	0.27	18.96	–
Stress (M)	PA (X)	-0.42	0.06	-7.00	<0.001	[-0.54, -0.30]			
Stress (M)	Support (S)	-0.39	0.05	-7.80	<0.001	[-0.49, -0.29]			
Stress (M)	X × S	0.15	0.05	3.00	0.003	[0.05, 0.25]			0.02
NSSI (Y)	Controls	0.09	0.04	2.25	0.026	[0.01, 0.17]	0.63	72.35	–
NSSI (Y)	PA (X)	-0.32	0.05	-6.40	<0.001	[-0.42, -0.22]			
NSSI (Y)	Support (S)	-0.28	0.05	-5.60	<0.001	[-0.38, -0.18]			
NSSI (Y)	Stress (M)	0.51	0.04	12.75	<0.001	[0.43, 0.59]			
NSSI (Y)	M × S	-0.14	0.05	-2.80	0.005	[-0.24, -0.04]			0.01

The analysis revealed that the interaction between PA and parental support (X×S) significantly predicted perceived stress (β=0.15, p=0.003, 95%CI=[0.05, 0.25]), contributing an incremental variance of 2% (ΔR²=0.02, p=0.003) beyond the main effects. Furthermore, the interaction between perceived stress and parental support (M×S) was a significant negative predictor of NSSI (β=−0.14, p=0.005, 95%CI=[−0.24, −0.04]), contributing an additional 1% to the explained variance (ΔR²=0.01, p=0.005).

The overall moderated mediation index was statistically significant (Index = -0.03, Boot SE = 0.01, Boot 95% CI = [-0.05, -0.01]), confirming that the indirect association of PA with NSSI through perceived stress is significantly moderated by the level of parental support. To clarify the practical significance of this moderation, conditional indirect associations were calculated at three levels of parental support (mean minus one standard deviation, mean, and mean plus one standard deviation), as detailed in **Table 6**.

**Table 6:** Conditional indirect associations of PA on NSSI via perceived stress at different levels of parental support.

Parental support level	Conditional indirect effect	Boot SE	Boot 95% CI
Low (M - 1SD)	-0.37	0.06	[-0.49, -0.25]
Medium (M)	-0.21	0.04	[-0.29, -0.13]
High (M + 1SD)	-0.10	0.04	[-0.18, -0.03]

As illustrated in **Table 6**, while the indirect association was significant across all levels, its magnitude progressively weakened as parental support increased, indicating that the mediational pathway is particularly robust for adolescents lacking familial support.

### 3.5 Simple slope analysis

To facilitate the interpretation of these interaction effects, simple slope analyses were performed at high (M + 1SD), medium (M), and low (M - 1SD) levels of parental support. As shown in **Table 7**, physical activity was significantly associated with lower perceived stress across all levels of parental support, but the association was most robust under conditions of low parental support (β = -0.57, p < 0.001) and attenuated as support increased. This positive interaction coefficient (β = 0.15) and the corresponding simple slopes indicate a compensatory mechanism. Rather than high parental support amplifying the benefits of PA, it implies that PA serves as a critical, primary coping mechanism precisely when external emotional support is scarce.

**Table 7:** Simple slope: PA -> perceived stress at levels of parental support.

Parental support level	β	SE	t	p	95% CI
Low (M - 1SD)	-0.57	0.08	-7.12	<0.001	[-0.73, -0.41]
Medium (M)	-0.42	0.06	-7.00	<0.001	[-0.54, -0.30]
High (M + 1SD)	-0.27	0.07	-3.86	<0.001	[-0.41, -0.13]

**Figure 2:**
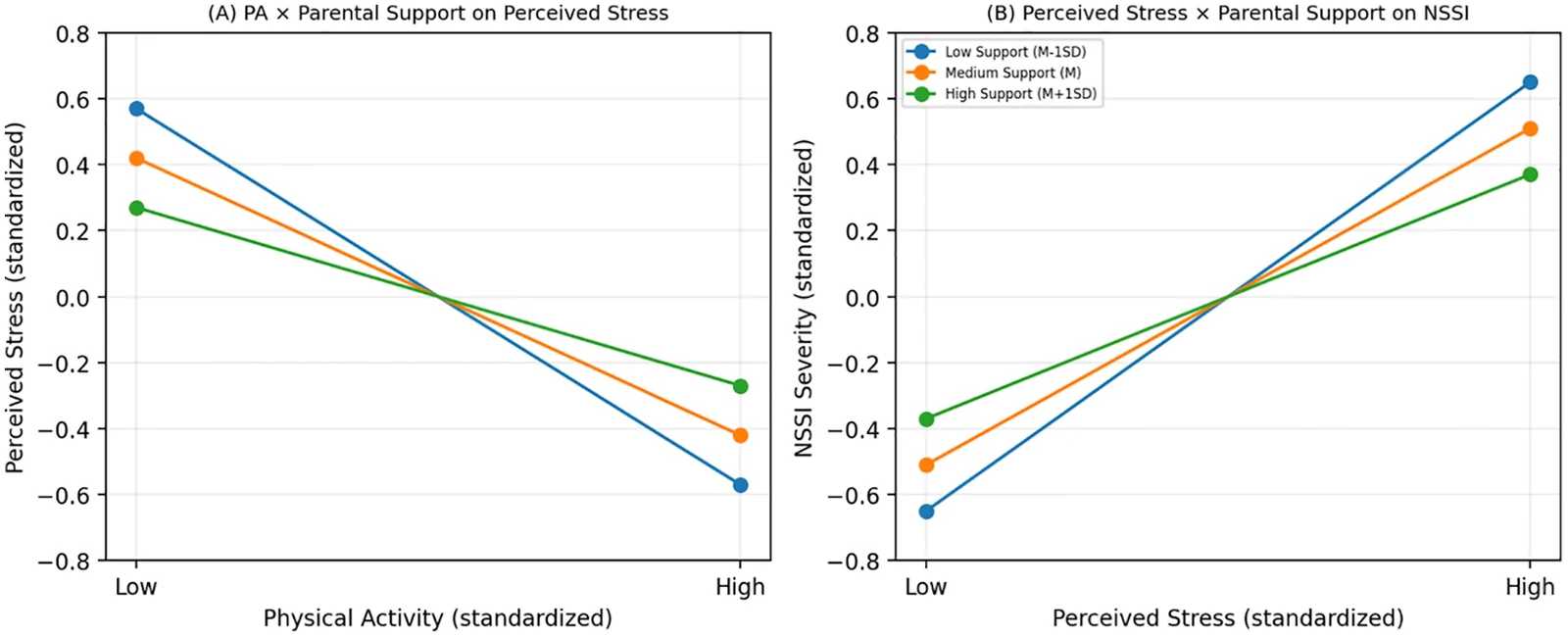
Interaction of physical activity and perceived stress with parental support. **(A)** Illustrates the interaction between PA and parental support on perceived stress. **(B)** Illustrates the interaction between perceived stress and parental support on NSSI.

As indicated in **Table 8**, the positive association between perceived stress and NSSI was significant across all support levels, but its predictive strength was significantly weakened in adolescents with high parental support (β = 0.37, p < 0.001) compared to those with low support (β = 0.65, p < 0.001). These dynamics are visually summarized in **Figure 2**.

**Table 8:** Simple slope: perceived stress -> NSSI at levels of parental support.

Parental support level	β	SE	t	p	95% CI
Low (M - 1SD)	0.65	0.06	10.83	<0.001	[0.53, 0.77]
Medium (M)	0.51	0.04	12.75	<0.001	[0.43, 0.59]
High (M + 1SD)	0.37	0.06	6.17	<0.001	[0.25, 0.49]

## 4 Discussion

The present study systematically explored the psychological indirect associations and boundary conditions underlying the association between physical activity (PA) and adolescent non-suicidal self-injury (NSSI). By validating a moderated mediation model, this research elucidates how PA is linked to lower NSSI risk through its association with lower perceived stress, and how this statistical pathway is contingent upon the quality of parental support. The findings offer a nuanced understanding of the psychological, social, and behavioral interactions that shape adolescent high-risk behaviors, moving beyond simple direct-effect models to reveal a more complex regulatory framework.

### 4.1 In-depth interpretation and dialogue with existing literature

#### 4.1.1 The protective association of physical activity on NSSI

The empirical results demonstrate that PA maintains a robust negative association with adolescent NSSI (35, 36). This finding is consistent with evidence that PA is associated with lower psychological distress and suicidality in adolescents (12, 36–38). It is also compatible with functional models of NSSI, which emphasize that self-injury is often maintained by affect regulation, experiential avoidance, and negative reinforcement processes (39–41). From this perspective, PA may offer an adaptive route for emotional discharge and physiological arousal regulation, thereby competing with the maladaptive regulatory function served by NSSI. This interpretation is supported by prior studies showing that exercise can reduce sensitivity to stress and improve affective well-being (16, 42, 43). The decomposition of effects revealed that the direct association between PA and NSSI accounted for 63.79% of the total statistical association. This large direct component warrants careful interpretation rather than being treated as residual noise. First, from a behavioral replacement perspective, structured PA may reduce idle and isolated time during which adolescents are more vulnerable to repetitive rumination, emotional cascades, and self-injurious urges (18, 39). Second, PA may partly substitute for the pain-offset or affect-regulation function of NSSI. Reviews of self-injury functions consistently show that the most common motive for NSSI is rapid relief from intolerable affective states (40, 41); vigorous or structured exercise may provide a non-destructive way to regulate arousal, tension, and mood (38, 42). Third, regular PA may strengthen physical self-concept, body-related self-efficacy, and perceived mastery (44, 45), which could counteract self-punishment, body dissatisfaction, and dissociative motives frequently reported in NSSI research (39–41). Thus, the direct pathway should be understood as a theoretically meaningful domain for future measurement rather than as an unsupported causal claim. At the same time, the total association between PA and NSSI observed in the current sample was large for an observational study in developmental psychopathology. Meta-analytic and review evidence on PA and mental health outcomes in youth generally indicates beneficial but variable effects (9, 12, 38). Therefore, the magnitude observed here should be interpreted cautiously. It may partly reflect the formula-based composite scoring matrix of the PARS-3, which multiplies intensity, duration, and frequency and may expand the variance distribution non-linearly. It may also reflect shared method variance and the cross-sectional nature of the data. Future studies using accelerometry, ecological momentary assessment, or longitudinal modeling are needed to obtain more conservative and temporally robust estimates.

#### 4.1.2 The mediating role of perceived stress: from physiological resilience to cognitive appraisal

Our analysis identifies perceived stress as a critical psychological bridge, accounting for 36.21% of the total statistical association of PA with NSSI. This finding is consistent with studies identifying stress as a proximal risk factor for repetitive NSSI (13, 19) and with theoretical models that link emotional escalation, rumination, and maladaptive self-regulation to self-injurious behavior (18, 39–41). According to the Cross-Stressor Adaptation Hypothesis, regular PA can act as a manageable physiological stressor that promotes more efficient stress-response regulation (16). Experience sampling and accelerometry evidence also suggests that PA can buffer the association between daily stress and negative affect during high-pressure periods (17). Within this framework, PA may be associated with lower NSSI not only by improving general fitness but also by reducing subjective stress appraisals before they intensify into emotional cascades. Nevertheless, the mediation was partial rather than complete. This result is important because it prevents an overly narrow interpretation of PA as merely a stress-reduction tool. Functional theories of NSSI suggest that self-injury can be motivated by multiple intrapersonal and interpersonal functions, including affect regulation, self-punishment, anti-dissociation, communication of distress, and interpersonal influence (39–41). Recent Chinese adolescent evidence also suggests that PA-NSSI associations may operate through psychological capital and relative deprivation (46), supporting the need to examine multiple mediators. Therefore, future research should examine additional mediators such as emotion dysregulation, rumination, body image, sleep quality, peer victimization, and psychological capital to clarify which mechanisms are most sensitive to PA-based interventions.

#### 4.1.3 The dual moderating role of parental support: compensatory and buffering effects

A major innovation of this study is the verification of parental support as a dual moderator within the mediation chain. Regarding the PA -> perceived stress pathway, simple slope analysis revealed a compensatory mechanism: the negative association between PA and perceived stress was stronger among adolescents with low parental support. This pattern aligns with COR theory (22) and resource substitution perspectives (23), which propose that when one important resource is scarce, alternative resources may become more salient for coping. In the present context, adolescents who lack a stable family-based emotional resource may rely more heavily on PA as an accessible behavioral resource for stress relief and self-regulation. This explanation is also consistent with evidence linking social support, self-efficacy, and adolescent PA participation (44, 47). Regarding the perceived stress -> NSSI pathway, parental support showed a buffering effect. Adolescents perceiving high levels of trust, communication, and emotional security from parents showed a weaker association between stress and NSSI. This result is directly consistent with the classic stress-buffering hypothesis, which argues that social support protects individuals from the harmful consequences of stress (20, 48). It also converges with longitudinal and family-based evidence showing that parent-adolescent relationship quality and family factors are important predictors of NSSI onset and maintenance (49, 50). In contrast, low parental support may leave adolescents with fewer interpersonal resources to reinterpret stressors, seek help, or inhibit impulsive self-harm. By integrating compensatory and buffering effects, the present model demonstrates that individual lifestyle resources and family relational resources operate interdependently rather than independently.

### 4.2 Theoretical significance

Theoretically, this research contributes to the existing body of knowledge by synthesizing psychological and social perspectives into a unified explanatory framework for adolescent NSSI. By validating the “PA →Perceived Stress →NSSI” moderated mediation pathway, we provide strong empirical support for the integration of the Cross-Stressor Adaptation Hypothesis and the Emotional Cascade Model, clarifying how physiological training translates into behavioral inhibition through cognitive mediation. Furthermore, by identifying the dual moderating role of parental support, this study refines the Stress-Diathesis Model, demonstrating that environmental protective factors can regulate both the generation of stress and its behavioral consequences (14, 15). This underscores the necessity of considering individual-environment interactions in developmental psychopathology and provides a more sophisticated application of ecological systems theory to the study of high-risk adolescent behaviors (11, 51). The revelation of the “compensatory effect” of PA in low-support environments particularly enriches our understanding of resilience and resource substitution among vulnerable populations.

### 4.3 Practical implications

The findings have several targeted implications for the prevention and intervention of adolescent NSSI in school and clinical settings. First, school systems should elevate the status of physical education from a mere curricular requirement to a core mental health intervention strategy. Promoting consistent, moderate-to-vigorous aerobic activities— ideally exceeding 150 minutes per week—can provide adolescents with a robust physiological buffer against the pressures of academic and social life (9, 52). Second, mental health services should prioritize stress management as a key intervention target, utilizing PA as a foundational tool to improve cognitive appraisal and emotional stability (13, 42). Third, the critical role of the family environment cannot be overstated in long-term prevention. Family-centered interventions should focus on enhancing parental emotional support, communication skills, and trust-building to create a lasting safety net. Importantly, recognizing the compensatory nature of physical activity provides actionable guidance for targeted interventions: for adolescents whose family support is currently inadequate or dysfunctional, school-based PA programs should be deliberately strengthened to serve as a substitute protective buffer, thereby compensating for the lack of familial resources and helping to interrupt self-injurious cycles (24, 49).

### 4.4 Limitations and future research directions

Despite the rigorous methodology and theoretical contributions, several critical limitations must be explicitly acknowledged to contextualize these findings and guide future inquiry. First, the cross-sectional nature of the data fundamentally precludes the determination of temporal precedence and causal relationships. Given the cross-sectional design, all mediation and moderation paths represent statistical associations rather than definitive causal trajectories. While we conceptualized PA as a predictor of reduced stress and NSSI, reverse causality is theoretically plausible: adolescents experiencing acute emotional distress, high perceived stress, or active NSSI episodes may intrinsically lack the motivation, physical energy, or social functioning to participate in physical activities. Future experimental or longitudinal tracking designs are strictly necessary to establish definitive causal directionality. Second, although statistical procedures (CFA and ULMF) suggested that common method bias did not severely distort our measurement model, the exclusive reliance on single-informant self-report measures remains a significant methodological constraint. Subjective reporting may artificially inflate the observed correlations between predictor and outcome variables. Future research would benefit from incorporating multi-method assessments, such as accelerometry for PA and clinical interviews or experience sampling methods for NSSI. Third, our sample spanned a broad developmental period (ages 12 to 18) and pooled both junior and senior high school students. Although our preliminary sensitivity analyses suggested stable correlational directions across these cohorts, the cognitive, emotional, and social capacities of adolescents undergo profound shifts during these formative years. Future models should explicitly explore developmental stage as a formal moderating factor to unpack potential age-specific mechanisms.

Fourth, the measurement instruments carry specific psychometric limitations. The PARS-3 relies heavily on subjective recall, and its formula-based composite scoring matrix may obscure the independent contributions of exercise intensity versus duration. Additionally, while the DSHI is validated for adolescent use, its 7-point frequency scale anchored at “more than 20 times” may be suboptimal for capturing the subtle nuances of low-to-moderate frequency NSSI within non-clinical community samples. Fifth, the study’s generalizability is geographically restricted to five schools in Sichuan Province, China. The dynamics of family support, academic stress, and physical activity may vary significantly across broader cultural, ethnic, or socio-economic backgrounds, necessitating broader cross-cultural validation. Finally, the near-collinearity between participant age and school grade (r=0.89) posed a statistical challenge. While we managed this by excluding grade from the final regression models to prevent the inflation of standard errors, this adjustment highlights the inherent difficulty of disentangling maturational age effects from educational context effects in school-based cohort studies.

## 5 Conclusion

Based on a quantitative analysis of 402 adolescents, this study demonstrates that physical activity is a robust protective factor significantly associated with lower non-suicidal self-injury. The relationship is partially mediated by perceived stress, which accounts for 36.21% of the total statistical association, indicating that PA is linked to lower NSSI risk by optimizing the individual’s stress-response system and cognitive appraisal processes. Furthermore, parental support plays a vital moderating role in this mediated process. It serves a compensatory function by enhancing PA’s stress-reduction benefits in lowsupport environments and acts as a safety net by attenuating the link between distress and self-harm. These results emphasize that the effective prevention of adolescent NSSI requires a holistic strategy that combines the promotion of active lifestyles with the cultivation of supportive, secure family relationships.

## Data Availability

The raw data supporting the conclusions of this article will be made available by the authors, without undue reservation.
