# Supplementary material for: The moderated mediation effect of physical activity on non-suicidal self-injury: the mediating role of perceived stress and the moderating role of parental support
Source: Front Psychiatry. 2026 Aug 19;17:1824693. doi: 10.3389/fpsyt.2026.1824693 (PMC13535768; doi:10.3389/fpsyt.2026.1824693)
Supplement: Supplementary file 1 [file SupplementaryFile1.docx]

# Supplementary Material

## Supplementary Material S1. Questionnaire Sources and Access Procedures

To clarify the source and acquisition route of the questionnaires used in the present study, we provide the following information. All instruments were obtained from published academic scale descriptions and previously validated applications rather than commercial distribution. They were used only for anonymous, non-commercial academic research after school approval and written informed consent from students and legal guardians.

The Deliberate Self-Harm Inventory (DSHI) was selected based on the original behavior-specific self-harm measurement publication by Gratz (25). Evidence from modified DSHI use in adolescent samples was also consulted (26). The present study used the published behavior categories and frequency-reporting logic; Chinese wording was checked by bilingual researchers against published Chinese adolescent NSSI studies to maintain conceptual equivalence.

The Physical Activity Rating Scale-3 (PARS-3) was obtained from the Chinese revised exercise-rating scale reported by Liang (27), which is widely used in Chinese student physical-activity research. The original three dimensions – intensity, duration, and frequency – were retained. The scoring method followed the established formula: intensity × (duration − 1) × frequency. During classroom administration, researchers provided time anchors such as standard 45-minute physical education classes and scheduled school running sessions to reduce recall bias.

The Perceived Stress Scale-10 (PSS-10) derives from Cohen et al.’s original Perceived Stress Scale (28). The study also drew on the psychometric review by Lee (29) and Chinese adolescent validation evidence by Liu et al. (30). The standard 10-item structure was retained; the Chinese wording followed previously published Chinese applications and was checked by the research team for age-appropriate comprehension before data collection.

The Parent Scale of the Inventory of Parent and Peer Attachment short form (IPPA-45 parent scale, 15 items) was selected based on the original IPPA framework by Armsden and Greenberg (31) and Chinese IPPA-R validation in junior students by Zhang et al. (32). The present study further evaluated its psychometric performance using confirmatory factor analysis in the current sample.

All questionnaires were administered collectively in classrooms by trained researchers using a standardized script. Teachers left the classroom during administration to reduce social desirability pressure. Completed questionnaires were checked immediately for obvious omissions, and data were entered using a double-entry procedure. Extreme physiologically implausible physical-activity reports were verified or excluded before analysis.
